# Genetic Variation in CCL5 Signaling Genes and Triple Negative Breast Cancer: Susceptibility and Prognosis Implications

**DOI:** 10.3389/fonc.2019.01328

**Published:** 2019-12-06

**Authors:** Jingxuan Shan, Aziz Chouchane, Younes Mokrab, Mohamad Saad, Salha Boujassoum, Rosalyn W. Sayaman, Elad Ziv, Noureddine Bouaouina, Yasmine Remadi, Sallouha Gabbouj, Jessica Roelands, Xiaojing Ma, Davide Bedognetti, Lotfi Chouchane

**Affiliations:** ^1^Department of Genetic Medicine, Weill Cornell Medicine, New York, NY, United States; ^2^Department of Microbiology and Immunology, Weill Cornell Medicine, New York, NY, United States; ^3^Laboratory of Genetic Medicine and Immunology, Weill Cornell Medicine-Qatar, Doha, Qatar; ^4^Faculta di Medicina e Chirurgia, Universita Cattolica del Sacro Cuero, Rome, Italy; ^5^Translational Genetics and Bioinformatics Section, Research Division, Sidra Medicine, Doha, Qatar; ^6^Qatar Computing Research Institute, Hamad Bin Khalifa University, Doha, Qatar; ^7^Department of Medical Oncology, National Center for Cancer Care and Research, Hamad Medical Corporation, Doha, Qatar; ^8^Department of Population Sciences, City of Hope, Duarte, CA, United States; ^9^Department of Laboratory Medicine at UCSF, San Francisco, CA, United States; ^10^Helen Diller Family Comprehensive Cancer Center at UCSF, San Francisco, CA, United States; ^11^Division of General Internal Medicine, Department of Medicine, Institute for Human Genetics at UCSF, San Francisco, CA, United States; ^12^Service de Cancérologie Radiothérapie, CHU Farhat Hached, Sousse, Tunisia; ^13^Laboratoire d'Immuno-Oncologie Moléculaire, Faculté de Médecine de Monastir, Université de Monastir, Monastir, Tunisia; ^14^Tumor Biology Section, Research Division, Sidra Medicine, Doha, Qatar

**Keywords:** *CCL5*, CCL5 signaling genes, triple negative breast cancer, prognosis, susceptibility

## Abstract

Triple-negative breast cancer (TNBC) accounts for ~15–20% of breast cancer (BC) and has a higher rate of early relapse and mortality compared to other subtypes. The Chemokine (C-C motif) ligand 5 (CCL5) and its signaling pathway have been linked to TNBC. We aimed to investigate the susceptibility and prognostic implications of genetic variation in CCL5 signaling genes in TNBC in the present study. We characterized variants in *CCL5* and that of six other CCL5 signaling genes (*CCND1, ZMIZ1, CASP8, NOTCH2, MAP3K21, and HS6ST3*) among 1,082 unrelated Tunisian subjects (544 BC patients, including 196 TNBC, and 538 healthy controls), assessed the association of the variants with BC-specific overall survival (OVS) and progression-free survival (PFS), and correlated CCL5 mRNA and serum levels with *CCL5* genotypes. We found a highly significant association between the ***CCND1* rs614367-TT** genotype (OR = 5.14; *P* = 0.004) and TNBC risk, and identified a significant association between the **rs614367-T** allele and decreased PFS in TNBC. A decreased risk of lymph node metastasis was associated with the ***MAP3K21* rs1294255-C** allele, particularly in **rs1294255-GC** (OR = 0.47; *P* = 0.001). *CCL5* variants (**rs2107538** and **rs2280789**) were linked to CCL5 serum and mRNA levels. In the TCGA TNBC/Basal-like cohort the ***MAP3K21* rs1294255-G** allele was associated with a decreased OVS. High expression of *CCL5* in breast tumors was significantly associated with an increased OVS in all BC patients, but particularly in TNBC/Basal-like patients. In conclusion, genetic variation in CCL5 signaling genes may predict not only TNBC risk but also disease aggressiveness.

## Introduction

Triple-negative breast cancer (TNBC) accounts for 15–20% of patients diagnosed breast tumors. TNBC tumors relapse early after standard chemotherapy treatments and often develop visceral metastases; it thus represents a subtype with the poorest prognosis among all breast cancers. Others and our group showed that Arab women might experience an early disease onset and a preponderance of aggressive breast cancer phenotypes (1). To some extent, high rates of TNBC may explain the poor prognosis of BC diagnosed at a younger age in Arab populations. Studies of Arab American women showed a similar BC pattern as Arab women living in the Arab world (2, 3), suggesting that a genetic component could be among the determining factors associated with TNBC onset.

Immunity plays a crucial role in TNBC biology (4). TNBC, and/or the basal-like subtype, is characterized by higher mutational burden and lymphocyte density as compared to other subtypes (and especially as compared with estrogen/progesterone receptor positive tumors) (5). In TNBC, the density of tumor infiltrating lymphocytes has been associated with favorable prognosis (6, 7). Recently, the immune checkpoint blockade Atezolizumab (monoclonal antibody against the protein programmed cell death-ligand 1) has been approved by the FDA for the treatment of metastatic TNBC based on the positive results of a phase III trial (8). Furthermore, post-treatment chemokine (C-C motif) ligand 5 (CCL5) increase has been associated with responsiveness to immunotherapy (Imiquimod) in breast cancer skin metastasis (9).

Our recent work demonstrated that CCL5, is strongly implicated in BC pathogenesis, particularly in TNBC progression (10). Hematopoietic CCL5 plays a pivotal role in recruiting immune cells into the tumors (11, 12). The circulating CCL5 level variations, observed in BC patients, could reflect a functional genetic variation in *CCL5*. Three Single Nucleotide Polymorphisms (SNPs) in *CCL5*, namely rs2107538, rs2280788, and rs2280789, are the most frequent SNPs associated with inflammatory diseases (13). Thus far, potential association between genetic variation in *CCL5* and breast cancer has not been investigated. Integrative genomics analysis, combining information from GWAS studies on breast cancer involving over 400,000 cases and over 400,000 controls, was performed by Hicks et al. (14) to determine whether genes containing SNPs associated with an increased risk of developing breast cancer are associated with TNBC. Twelve out the 34 large-effect SNPs associated with TNBC are located within genes involved in the JNK, p38 MAPK, NF-κB, and cAMP/PKA signaling pathways, all of which regulate CCL5 levels in immune cells (15–17). These findings prompted us to hypothesize that through their effect on circulating CCL5 levels, functional polymorphism in both *CCL5* and CCL5 signaling genes could be associated with TNBC.

In addition to the three SNPs of *CCL5*, we selected six SNPs from the twelve associated with TNBC and involved in the JNK, p38 MAPK, NF-κB, and cAMP/PKA signaling pathways. These SNPs are tagged to *CCND1, ZMIZ1, CASP8, NOTCH2, MAP3K21, and HS6ST3*. These genes encode proteins which regulate CCL5 expression or themselves are regulated by CCL5. *CCND1* (cell-cycle regulator cyclin D1) encodes a cyclin protein that is critical for the cell cycle. The CCL5/STAT/CCND1 signaling pathway plays an important role in the crosstalk between epithelial cells and immune cells (18). *ZMIZ1* (zinc finger MIZ-type containing 1) encodes a transcription factor which is a member of the Protein Inhibitor of Activated STAT (PIAS)-like family of coregulators. Zmiz1 is important for T-cell development and involved in NOTCH signaling (19), by which it could regulate CCL5 expression (20). *CASP8* encodes the Caspase 8 protein which plays a major role in cell apoptosis and regulates NF-κB signaling (21). The functional association between *CASP8* and CCL5 levels, secreted by immune cells, was shown in a knockout mice model (22). *NOTCH2* encodes a member of NOTCH transmembrane receptor family. Dysregulation of NOTCH signaling was involved in several diseases, including BC (23). CCL5 expression is activated by NOTCH signaling in the tumor microenvironment, both in cancer cells (20) and tumor infiltrating lymphocytes (24, 25). *MAP3K21* gene encodes a member of the MAPK pathway (also known as MLK4). MAP3K21 serves as an activator of NF-κB signaling (26); a major pathway for inducing CCL5 expression (27). *HS6ST3* encodes a member of Heparan sulfate (HS) involved in several cellular and molecular processes, including cell proliferation and differentiation (28). By regulating IGFR1 expression, HS6ST3 could affect CCL5 expression (29, 30).

Based on the abundant evidence of the role of CCL5 in TNBC, we evaluated, in this study, the association of 9 SNPs, reflecting the genetic variation in *CCL5* signaling genes, with TNBC susceptibility and prognosis.

## Materials and Methods

### Patients and Controls

A total of 1,082 unrelated subjects with high quality of genomic DNA, comprising 544 breast cancer patients, including 196 TNBC, and 538 healthy controls, were included in this study. Controls and patients were selected from the same ethnic group living in the middle coast of Tunisia. Only patient/control subjects who have ancestors up to three generations back who were natives of Tunisia and have lived for at least 10 years in Tunisia were included in the study. The participation rate for patients and controls exceeded 90 and 75%, respectively.

All patients included in this study had primary breast cancer, with unilateral breast tumors and no family history of the disease. The diagnosis of cancer was confirmed by histopathological analyses. The mean age of patients was 48.8 ± 10.9 years. After completion of treatment, patients had regular visits every 3–4 months in the first 2 years, every 6 months in the following 3 years and annually thereafter. At each visit patients were checked for symptoms and undergo a physical examination, mammography, chest X-ray and abdominal ultrasound were performed annually. During follow up, both locoregional and distant tumor recurrence were diagnosed as relapse based on clinical, radiological and histological findings. The median follow-up was 55 months (range, 1–175 months). Data on each patient were collected from the department of Radiation Oncology of Sousse Hospital (Sousse, Tunisia) between 2002 and 2016. A detailed description of the clinical/pathological characteristics is summarized in Table 1.

**Table 1:** Clinicopathologic characteristics of the breast carcinoma patients.

**Characteristics**	**Cases**
**Age range**	23–91
**Age at diagnosis (mean** ± **SD)**	48.8 ± 10.9
Menopausal status^*^	
Pre-menopausal	235
Post-menopausal	263
Tumor size^*^	
T_0−2_	254
T_3−4_	157
**Regional lymph node involvement**	
Positive (N_1−3_)	208
Negative (N_0_)	306
Histological grade^*^	
SBR_1−2_	322
SBR_3_	172
**Pathological lymph node involvement**	
Positive (pN_1−3_)	323
Negative (pN_0_)	190
**Distant metastasis**	
Positive (M_1_)	79
Negative (M_0_)	465
Relapse^*^	
Positive	88
Negative	455
**Death record**	
Positive	11
Negative	533
Estrogen receptor (ER) status^*^	
Positive	277
Negative	222
Progesterone receptor (PR) status^*^	
Positive	233
Negative	268
ER/PR status^*^	
ER^+^/PR^+^	206
ER^+^/PR^−^	70
ER^−^/PR^+^	24
ER^−^/PR^−^	198
**Triple negative**	
Yes	196
No	348

* *Some data are missing*.

A total of 538 healthy women with a mean age of 48 ± 14 years were recruited as controls. They are typically either outpatients seen for vaccination or blood donation or friends or non-biological relatives of cancer patients with no evidence of any personal or family history of cancer (or other serious illness). Samples from healthy controls were collected consecutively between 2004 and 2016 and were age–group matched to the cases.

After a written consent was obtained, each participant (patient/control) had a blood sample taken and was interviewed using a questionnaire. The questionnaire has been tested, standardized and endorsed by the oncologists involved in the recruitment of patients with breast cancer. This questionnaire included a collection of epidemiological and lifestyle data and a “genetic” part examining consanguinity of parents and familial history with detailed data on all first and second-degree relatives with breast cancer (age, disease status). Detailed clinical-pathologic characteristics of the tumor are included as well. For all patients with breast cancer, early treatment response was assessed at 3- and 6-months post-treatment and graded as complete, or partial or poor/disease progression. The blood samples from patients were taken before initiation of any treatment.

Approval for the study was given by the Weill Cornell Medicine-Qatar Ethics Committee. Both patients and controls gave their written consent to participate in the study.

TCGA Affymetrix Human SNP 6.0 array genotyping calls of patient germline DNA were downloaded from NCI's Genomic Data Commons (https://gdc.cancer.gov). SNPs were filtered to eliminate >5% missing genotypes, and individuals with >5% missing genotypes were also removed. Individuals were clustered into ancestry groups using principal components analysis and PAM clustering, and samples which had heterozygosity values that were >3 standard deviations from the mean heterozygosity from the same ancestral populations were also eliminated. Only one sample per individual were included, with blood-derived samples and samples with higher call rates preferentially retained. SNPs that deviated from the expectation under Hardy-Weinberg equilibrium (*p* < 1 × 10^−6^) in analysis of the largest ancestral group were also dropped (though SNPs previously associated with any cancer as reported in the GWAS catalog with *p* < 5 × 10^−8^ were not eliminated since they may deviate from HWE in cancer cases). SNPs with minor allele frequency, MAF <0.5% were excluded, and duplicate SNPs with identical genomic first positions were removed. Palindromic SNPs were excluded prior to stranding. Finally, we checked for close relative pair-based analyses in PLINK1.9 and dropped one of each of these relative pairs. Variants were imputed through Minimac 3 selecting the panel of 1000 Genome Phase 3v5 using mixed population. The final dataset includes 163 Basal-like/TN breast cancer patients with available overall survival information, which were used in the present analysis. Annotated Germline TCGA Exome data (31) were used to retrieve CCR5 delta-32 information.

### SNP Selection and Genotyping

We selected the three of the most studied SNPs of *CCL5* located in the promoter region of the *CCL5* gene. We also selected six SNPs from breast cancer GWAS studies (14). The six SNPs containing genes are all involved in signaling pathways that can regulate CCL5 levels. The SNP information is summarized in Table S1. Genotyping was performed using the TaqMan® SNP genotyping assays on Applied Biosystems QuantStudio 6 Flex Real-Time PCR System (Foster City, California, United States).

### Enzyme-Linked Immunosorbent Assay (ELISA)

A specific ELISA (Human CCL5/RANTES Quantikine ELISA Kit, R&D Systems, Minneapolis, Minnesota, United States) was used to determine CCL5 levels in serum before cancer treatment with a calorimeter (CLARIOstar, BMG LABTECH, Ortenberg, Germany) at a wavelength of 405 nm. Three hundred-sixteen breast cancer patients whose serum were available, including 128 TNBC patients, were tested in this study.

### *In silico* Analysis of *CCL5* Gene Expression

The allele specific gene expression was assessed at Genotype-Tissue Expression Portal (GTExPortal) (https://gtexportal.org/home/) (32) and eQTL Catalog (https://eqtl.onderzoek.io) (33). The cancer survival analysis with TCGA data was performed using UCSC XENA (https://xena.ucsc.edu) and for that with the gene expression array data was performed using KM plotter (http://kmplot.com) (34). Correlation between CCL5 and markers of stemness (cancer stem cells and normal stem cells) and epithelial to mesenchymal transition (EMT) markers, was performed by using a normalized RNA matrix, described elsewhere (35). The selection of markers was based on current literature (36).

### RNA Extraction and qPCR Quantification

RNA was extracted from frozen blood using PAXgene Blood RNA Kit (Qiagen, Germantown, Maryland, United States) using the following protocol (37). 300 μl of blood sample was dispensed into 830 μl PAXgene reagent. The mixture was incubated for overnight at room temperature and spun for 3 min at 5,000 g. After washing with 500 μl RNase-free water, the pellet was resuspended in 350 μl Buffer BR1 and incubated with 300 μl Buffer BR2 (binding buffer) and 40 μl proteinase K for 10 min at 55°C in a shaker-incubator at 1,400 rpm. The lysate was transferred into a PAXgene Shredder spin column placed in a 2 ml processing tube and centrifuged for 3 min at 18,000 g. The flow-through fraction was mixed with 350 μl ethanol, transferred to a PAXgene RNA spin column and centrifuges for 1 min at 18,000 g. After washing the column with Buffer BR3 (washing buffer I), samples were incubated with 80 μl of DNase I incubation mix (10 ul DNase I with 70 ul Buffer RDD) for 15 min at room temperature. PAXgene RNA spin columns were washed with Buffer BR3 and 2 times of Buffer BR4 (Washing buffer II). RNA was eluted with 40 μl Buffer BR5 (elution buffer).

Total RNA was reverse-transcribed into cDNA using SuperScript II Reverse Transcriptase (Invitrogen, Carlsbad, California, United States) following the manufacturer's protocol. *CCL5* mRNA levels were measured by qPCR with a GoTaq® 2-Step RT-qPCR System (Promega, Madison, Wisconsin, United States) for SYBR Green-based detection. The qPCR primers for *CCL5* are 5′-CCAGCAGTCGTCTTTGTCAC-3′ and 5′-CTCTGGGTTGGCACACACTT-3′. The *HPRT1* gene was used as a reference and primer sequences are 5′-TGACACTGGCAAAACAATGCA-3′ and 5′-GGTCCTTTTCACCAGCAAGCT-3′. Two hundred and twenty-six mRNA samples were tested in this study.

### Statistical Analyses

Hardy-Weinberg equilibrium in both patient and control groups were tested using the Chi-square test. According to the general genotype model, risk association between the genotypes and breast cancer susceptibility and tumor characteristics were estimated by a crude odds ratio (OR) with 95% confidence intervals (95% CI) using the unconditional logistic regression analysis with genotypes coded additively (as 0, 1, or 2 copies of one of the alleles). The association was then adjusted for age and menopausal status. A *P*-value of <0.05 was required for statistical significance.

The statistical analysis was performed using the Epi-Info statistical program (version 5.01; CDC, Atlanta, GA, USA). Breast cancer specific overall survival (OVS) was defined as the time from the date of diagnosis to death from breast cancer or to last contact. Progression-free survival (PFS) was defined as the time from the date of diagnosis to distant metastasis, relapse and death from BC or to last contact. The survival curves were plotted according to Kaplan and Meier. Differences between groups were calculated by the log rank test. For PFS survival of TNBC patients between rs614367 risk allele carriers and non-risk allele carriers, a multivariate Cox Regression analysis was performed for age, menopausal status, tumor stage, SBR grade, lymph node status and pathologic classification using SPSS statistic software (Version 25.0, IBM Corporation).

## Results

### Genetic Variation in *CCL5* Signaling Genes as Risk Factors for Breast Cancer and for TNBC

Genotype distribution of all 9 SNPs in controls did not deviate from Hardy-Weinberg equilibrium (Table S2, *P* > 0.05). The **rs2280788-C** allele is very rare in our study population compared to that found in the 1000 Genome and HapMap. The linkage disequilibrium (LD) between *CCL5* rs2107538 and *CCL5* rs2280789 was calculated using SHEsis (38). In controls, D′ is 0.763 and r^2^ is 0.407; in cases, D′ is 0.906 and r2 is 0.488. These results indicate a strong LD between these two loci. However, the allele frequencies between these two loci are quite different (Table 2). Therefore, we continued the analysis of these two loci separately.

**Table 2:** Association of 9 SNPs of the CCL5 and CCL5 signaling pathway with breast carcinoma^a^ risk.

**SNP**	**Gene**	**Genotype**	**Risk allele frequency**		**Heterozygotes^b^**		**Homozygotes^c^**		**Per risk allele**	
			**Cases (*n* = 544)**	**Controls (*N* = 538)**	**OR (95%CI)**	***P***	**OR (95%CI)**	***P***	**OR (95%CI)**	***P***
rs2107538	*CCL5*	C/T	0.206	0.149	**1.36 (1.04–1.77)**	0.025^**^	**2.74 (1.337–5.60)**	0.004^**^	**1.47 (1.17–1.84)**	**0.0007**
rs2280788	*CCL5*	G/C	0.009	0.002	2.67 (0.70–10.11)	NS	3.0 (0.12–73.81)	NS	3.32 (0.91–12.09)	0.054
rs2280789	*CCL5*	A/G	0.132	0.109	1.13 (0.85–1.51)	NS	**5.52 (1.12–23.69**)	0.019^*^	1.25 (0.96–1.62)	0.092
rs614367	*CCND1*	C/T	0.13	0.099	1.18 (0.87–1.59)	NS	**3.64 (1.19–11.16)**	0.016^**^	**1.38 (1.02–1.71)**	**0.032**
rs704010	*ZMIZ1*	C/T	0.321	0.288	1.01 (0.79–1.30)	NS	1.51 (0.99–2.31)	NS	1.15 (0.96–1.38)	NS
rs1045485	*CASP8*	G/C	0.136	0.124	1.05 (0.79–1.41)	NS	1.37 (0.62–3.03)	NS	1.10 (0.85–1.41)	NS
rs1124933	*NOTCH2*	G/A	0.319	0.339	0.80 (0.62–1.03)	NS	0.90 (0.61–1.31)	NS	0.90 (0.75–1.07)	NS
rs1294255	*MAP3K21*	G/C	0.425	0.411	1.0 (0.77–1.31)	NS	1.09 (0.78–1.52)	NS	1.04 (0.88–1.23)	NS
rs1924587	*HS6ST3*	G/C	0.427	0.369	**1.33 (1.02–1.74**)	0.032^*^	**1.52 (1.07–2.17)**	0.021^**^	**1.26 (1.06–1.49)**	**0.009**

*CI, confidence interval; OR, odds ratio*.

a *Including all breast carcinoma patients*.

b *Heterozygotes compared to homozygotes of reference allele*.

c *Homozygotes of risk allele compared to homozygotes of reference allele*.

*The chi-square test with was used to determine whether significant differences (P-value) were observed when patient group was compared with control subjects. Significant P-values are in bold cases*.

** *The association remains significant after age and menopausal status adjustment*.

* *Not significant after age and menopausal status adjustment*.

A significant increase in the ***CCL5* rs2107538-T** and ***HS6ST3* rs1924587-C** allele frequencies was observed in the patient group compared to the controls (*P* = 0.0007 and 0.009, respectively, Table 2). The OR of breast cancer associated with the ***CCL5* rs2107538-T/T** genotype was 2.74 (*P* = 0.004) and that of ***HS6ST3* rs1924587-C/C** genotype was 1.52 (*P* = 0.021). The allelic frequency of the ***CCND1* rs614367-T** was higher in patients with breast cancer compared to control subjects resulting in a significantly positive OR associated with this allele (OR = 1.38, *P* = 0.032). This association was found to be specifically confined to the ***CCND1* rs614367-T/T** homozygous genotype (OR = 3.64, *P* = 0.016). In addition, a significant association was found between breast cancer and the ***CCL5* rs2280789-G/G** homozygous genotype (OR = 5.52, *P* = 0.019). After adjustment for the potential confounders, including age and menopausal status, the association of ***CCL5* rs2107538-C/T** genotype (*P* = 0.034), ***CCL5* rs2107538-T/T** genotype (*P* = 0.011), ***CCND1* rs614367-T/T** genotype (*P* = 0.021) and ***HS6ST3* rs1924587-C/C** genotype (*P* = 0.019) with BC risk remained significant.

In order to identify a specific association between certain *CCL5* signaling pathways and TNBC, we stratified the 544 patients with breast cancer according to the breast cancer subtype, namely TNBC and hormone receptor positive breast cancer (HRBC). A highly and significant increase in risk of TNBC compared to controls, but not of HRBC, was observed in subject carriers of the ***CCND1* rs614367-T/T** homozygous genotype (OR = 5.14, *P* = 0.004, Table 3 and Table S3). Among the 9 SNPs, only the ***CCND1* rs614367-T/T** genotype showed a strong and specific association with TNBC.

**Table 3:** Association of 9 SNPs of the CCL5 and CCL5 signaling pathway with triple negative breast cancer risk.

**SNP**	**Gene**	**Genotype**	**Controls**	**Cases**	**Heterozygotes^a^**		**Homozygotes^b^**		**Per risk allele**	
			***N* = 538**	***N* = 196**	**OR (95%CI)**	***P***	**OR (95%CI)**	***P***	**OR (95%CI)**	***P***
rs2107538	*CCL5*	CC	389	125	1.42 (0.99–2.03)	0.055^*^	2.26 (0.89–5.75)	0.079^*^	**1.45 (1.07–1.95)**	**0.015**
		CT	138	63						
		TT	11	8						
rs2280788	*CCL5*	GG	535	192	**4.98 (0.91–27.41)**	0.041^*^	2.49 (0.05–125.72)	NS	4.94 (0.9–27.07)	NS
		GC	3	4						
		CC	0	0						
rs2280789	*CCL5*	AA	423	148	1.11 (0.75–1.65)	NS	**5.72 (1.04–31.53)**	0.024^*^	1.254 (0.88–1.78)	NS
		AG	113	44						
		GG	2	4						
rs614367	*CCND1*	CC	435	148	1.22 (0.81–1.83)	NS	**5.14 (1.49–17.82)**	0.004^**^	**1.48 (1.04–2.09)**	**0.027**
		CT	99	41						
		TT	4	7						
rs704010	*ZMIZ1*	CC	271	94	1.02 (0.72–1.44)	NS	1.54 (0.88–2.69)	NS	1.16 (0.9–1.49)	NS
		CT	224	79						
		TT	43	23						
rs1045485	*CASP8*	GG	416	151	0.97 (0.64–1.46)	NS	1.5 (0.55–4.13)	NS	1.06 (0.75–1.5)	NS
		GC	111	39						
		CC	11	6						
rs1124933	*NOTCH2*	GG	233	96	0.73 (0.51–1.03)	NS	1 (0.64–1.57)	NS	0.93 (0.73–1.19)	NS
		GA	241	72						
		AA	64	28						
rs1294255	*MAP3K21*	GG	192	76	0.83 (0.72–1.19)	NS	0.99 (0.63–1.56)	NS	0.97 (0.77–1.23)	NS
		GC	245	80						
		CC	101	40						
rs1924587	*HS6ST3*	GG	216	63	**1.51 (1.05–2.17)**	0.025^*^	1.13 (0.67–1.91)	NS	1.15 (0.91–1.46)	NS
		GC	243	107						
		CC	79	26						

*CI, confidence interval; OR, odds ratio*.

a *Heterozygotes compared to homozygotes of reference allele*.

b *Homozygotes of risk allele compared to homozygotes of reference allele*.

*The chi-square test was used to determine whether significant differences (P-value) were observed when TNBC patient group was compared with control subjects. Significant P-values are in bold cases*.

** *The association remains significant after age and menopausal status adjustment*.

* *Not significant after age and menopausal status adjustment*.

The ***CCL5* rs2107538-T** allele was significantly associated with both TNBC and HRBC but the OR associated with HRBC was higher and more significant (OR = 1.45, *P* = 0.015 in TNBC compared to OR = 1.80, *P* = 0.0001 in HRBC). An increased risk of HRPC was found associated with the **rs1924587-C** allele (OR = 1.33, *P* = 0.006) and was confined to the homozygous **C/C** genotype (OR = 1.7, *P* = 0.012). A slightly significant association with TNBC was also observed in carriers of the **heterozygous G/C** genotype (OR = 1.51, *P* = 0.025).

Both ***CCL5* rs2280788-GC** and ***CCL5* rs2280789-GG** showed a slightly significant association with TNBC (OR = 4.98, *P* = 0.041; OR = 5.72, *P* = 0.024, respectively). Carriers of the **rs2280789-G** allele had a marginal increase in the risk for HRBC (OR = 1.36, *P* = 0.048).

After adjustment for the potential confounders, including age and menopausal status, the association of ***CCND1* rs614367-T/T** genotype (*P* = 0.015) with TNBC risk and both of the association of ***CCL5* rs2107538-T/T** genotype (*P* = 0.004) and ***HS6ST3* rs1924587-C/C** genotype (*P* = 0.004) with HRBC risk remained significant, and the association of ***CCL5* rs2107538-C/T** genotype (*P* = 0.049) with HRBC risk was suggestive.

### Prognostic Significance of Genetic Variation in the *CCL5* Signaling Pathway

Table S4 lists the clinical and pathological characteristics. The distribution of the clinical and pathological markers was in agreement with previously reported data, indicating that our cohort was representative of breast cancer patients. Progression-free survival (PFS) rates were estimated and compared by univariate analysis based on the clinical and pathological parameters. Significant associations were found for: clinical tumor size, lymph node status, and tumor grading with PFS. No significant differences were observed for age.

We investigated the potential association of 9 SNPs associated with the *CCL5* signaling pathway and the clinical and pathological characteristics of breast cancer in our cohort. As shown in Tables S5, S6, the **rs1294255-C** allele was found more frequently in patients with no lymph-node metastasis; this resulted in a significantly negative OR associated with heterozygous **rs1294255-G/C** (OR = 0.47; *P* = 0.001 compared to **GG** genotype).

**Figure 1:**
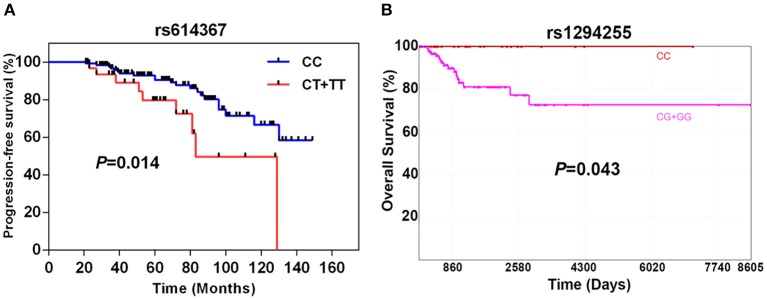
Breast cancer-specific progression-free survival of 196 TNBC patients according to the presence or absence of the ***CCND1* rs614367-T** allele **(A)** and specific 10-years overall survival of 163 TNBC patients from the TCGA dataset according to the presence or absence of ***MAP3K21* rs1284255-G** allele **(B)**.

As shown in Figure 1A, the progression-free survival (PFS) was significantly shorter in the group of TNBC patients carrying the ***CCND1* rs614367-T** allele. The estimated 5- and 10-years PFS rates in the groups of TNBC patients carrying or not the ***CCND1* rs614367-T** allele were, respectively, 78 and 50% vs. 92 and 66% (log rank test, *P* = 0.014). Furthermore, a multivariate analysis was performed for age, menopausal status, tumor stage, SBR grade, lymph node status and pathologic classification. As shown in Figure S1, the survival rates showed a difference trend between patients with and without the ***CCND1* rs614367-T** allele, but the difference did not reach statistical significance (*P* = 0.359).

The limited number of breast cancer specific death events in our cohort hampered the search for a potential association between the 9 SNPs and OVS. The analysis of the TCGA dataset, which included 163 TNBC/Basal-like patients with available OS and genotype information, revealed a significant association between the ***MAP3K21* rs1294255-G** allele and OVS. As shown in Figure 1B, the OVS was significantly shorter in the group of TNBC/Basal-like patients carrying the ***MAP3K21* rs1294255-G** allele. The estimated 10-years OVS in the groups of TNBC patients with or without the ***MAP3K21* rs1294255-G** allele was <80 and 100%, respectively (log rank test, *P* = 0.043). Similarly, *CCL5* rs2107538-T allele showed a suggestive association with reduced OVS in TNBC patients (Figure S2). No statistically significant associations with OS were observed for the other seven polymorphisms.

### Functional Implications for Breast Cancer and TNBC Risk Variants Associated With *CCL5* Expression

**Figure 2:**
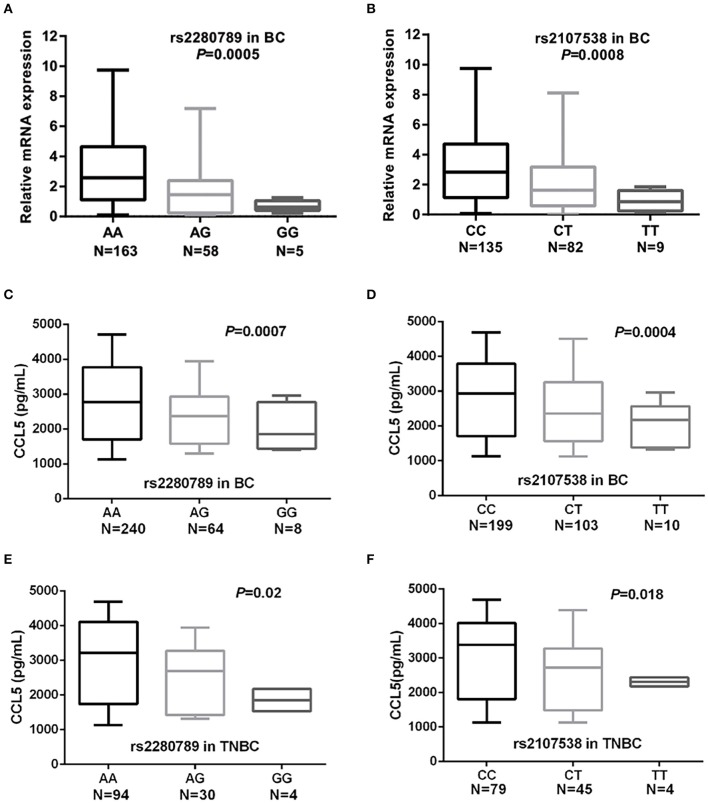
Quantification of *CCL5* mRNA levels in blood cells and serum CCL5 in of breast cancer and TNBC patients*. CCL5* mRNA levels in blood cells of breast cancer patients according to the genotypes of ***CCL5* rs2280789 (A)** and ***CCL5* rs2107538 (B)**. CCL5 serum levels in 312 patients with breast cancer and in 128 patients with TNBC according to the genotypes of ***CCL5* rs2280789 (C,E)** and ***CCL5* rs2107538 (D,F)**.

To assess whether the 9 SNPs were associated with changes in the expression of *CCL5* and/or their tagged genes, we performed eQTL (expression quantitative trait loci) analyses using GTEx and NESDA and NTR data. In a dose-dependent manner, the breast cancer risk alleles ***CCL5* rs2280789-G** and ***CCL5* rs2107538-T** were predictors of low levels of *CCL5* expression in whole blood and lymphocytes of GTEx dataset (*P* = 7 × 10^−6^ and 6 × 10^−8^, respectively) (Figures S3A,B). The **rs1294255-G** allele was associated with low levels of *MAP3K21* expression (Table S7). The association between *CCL5* variants and *CCL5* expression was confirmed by quantifying *CCL5* mRNA in 226 blood samples from our study population. qPCR analysis suggested that ***CCL5* rs2280789-G** and ***CCL5* rs2107538-T** alleles associated with low levels of *CCL5* mRNA (Figures 2A,B).

The CCL5 levels in serum before cancer treatment were correlated with patients' ***CCL5*** genotypes. As shown in Figures 2C,D, the breast cancer risk alleles ***CCL5* rs2280789-G** and ***CCL5* rs2107538-T** were highly associated in a dose-dependent manner with low levels of CCL5 in sera of patients with breast cancer (all patients without subtype stratification). Similar findings were observed in sera of TNBC patients (Figures 2E,F). The size effect of ***CCL5* rs2107538** was higher in patients with TNBC than that observed in all patients with breast cancer. No significant difference in CCL5 levels was found between sera of control subjects and that of cancer patients (Figure S4).

Taken together, these findings suggest a potential impact of *CCL5* expression levels on disease progression and clinical outcome. We addressed this hypothesis by assessing the correlation between *CCL5* expression in breast tumors and disease survival using the TCGA dataset. As shown in Figure S5A, high levels of *CCL5* expression predict a better survival in breast cancer patients (all subtypes included). This correlation did not reach statistical significance in cases with TNBC (likely duo to the limited sample size), but a trend toward higher survival rates was seen for TNBC tumors expressing high levels of *CCL5* (Figure S5B). Further, we used the KM plotter platform (including a higher number of samples, i.e., the ones for which gene expression array were available either from TCGA or deposited in GEO) to evaluate an association between *CCL5* expression and breast cancer and TNBC patients' survival. High levels of *CCL5* expression correlated with a highly significant increase effect for TNBC patients' survival (Figure S5D). The estimated 5-years survival probabilities in the TNBC tumors expressing high or low levels of CCL5 were 0.88 and 0.63, respectively (log rank test, *P* = 0.00045). A similar observation was made when the analysis was performed on the unstratified breast cancer group (Figure S5C); here *CCL5* expression had a less significant effect on the survival probabilities and the hazard ratio (HR) was higher compared to that observed in cases with TNBC (0.66 vs. 0.30). The estimated 5-years survival probabilities in the unstratified breast cancer tumors expressing high or low levels of CCL5 were 0.90 and 0.78, respectively (log rank test, *P* = 0.0093). Notably, the levels of *CCL5* expression had no significant effect on non-TNBC patient survival (data not shown) in line with the predominant prognostic effect of tumor infiltrating lymphocytes observed prevalently in TNBC.

**Figure 3:**
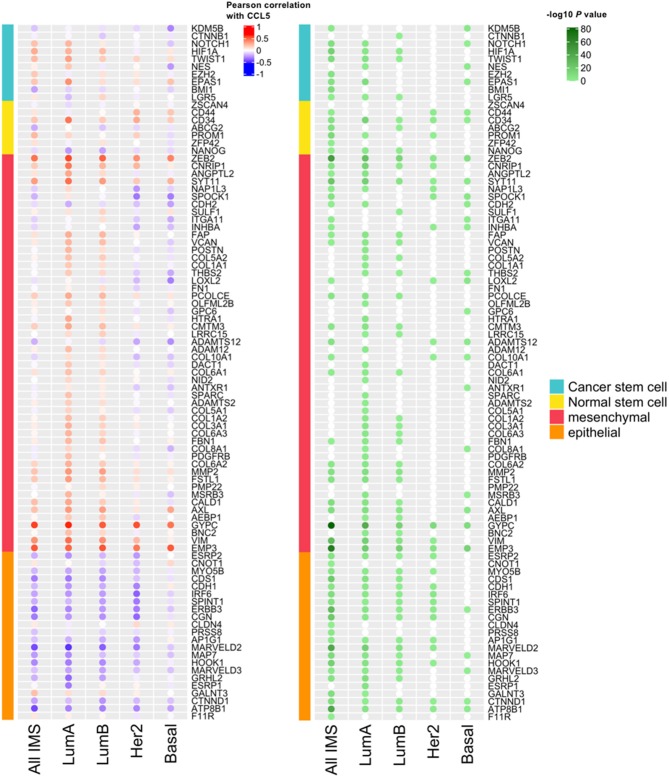
The correlation between *CCL5* expression and the expression of markers of stemness and Epithelial-Mesenchymal Transition (EMT) markers. IMS, intrinsic molecular subtype; Lum, luminal.

We next assessed in the TCGA cohort the potential association between CCL5 expression and markers of stemness and epithelial to mesenchymal transition (EMT) markers. As shown in Figure 3, CCL5 expression correlates at some extent with markers of stemness (cancer and normal stem cells) and with EMT markers in the analysis including all the intrinsic molecular breast cancer subtypes. However, such correlations were found particularly weak in the case of basal-like subtype, in which CCL5 was inversely correlated with mesenchymal markers. This might be explained by the pleiotropic role of CCL5, from one side being involved in TNBC pathogenesis, and from the other side mediating protective anti-tumor immunity via a positive immunologic loop resulting in lymphocyte recruitment through the interaction with its ligand CCR5 (co-expressed, together with CXCR3 by activated lymphocytes and NK cells). The inverse correlation between mesenchymal markers and CCL5 in the TNBC/basal-like subtype is also in line with the protective role of CCL5 observed in this group of tumors.

We also assessed the survival impact of a functional deleterious germline polymorphisms of the CCL5 receptor CCR5 (Δ32 polymorphisms) in the TCGA cohort. The CCR5-Δ32 polymorphism consists of a 32-base deletion encoding a truncated protein, which is not expressed on the cell surface (11) and is strongly associated with HIV resistance (in the homozygous status) or disease progression (in the heterozygous status) (39). No differences or trends were observed in term of overall survival, either in the entire BC cohort or in the basal-like cohort (Figure S6), when patients were stratified according to the CCR5-Δ32 status.

## Discussion

Given their crucial role in inflammatory diseases, chemokines were the subject of extensive study for several decades. Their role in recruiting immune cells to the tumor microenvironment made them an attractive potential target for cancer treatment. Among chemokines, CCL5 is relatively well-studied in cancers including breast cancer (40). However, knowledge of CCL5 implications with regard to cancer susceptibility remains elusive.

In the present study, we assessed the BC susceptibility of *CCL5* polymorphisms, including **rs2107538**, **rs2280788**, and **rs2280789**. The BC susceptibility locus ***CCL5* rs2107538** in our cohort was significantly associated with the risk for both TNBC and HRBC; the other breast cancer susceptibility locus **rs2280789** was associated with an increased risk for TNBC but only had a marginal effect on HRBC, while rs2280788 had a weak association with TNBC risk. We also assessed BC susceptibility of 6 SNPs in CCL5 signaling genes and found that ***CCND1* rs614367** was associated specifically with TNBC risk. ***HS6ST3* rs1924587** was preferentially associated with HRBC risk. ***MAP3K21* rs1294255** was associated with lymph-node invasion of breast tumors. For certain SNPs, we observed the association between BC and the allele risk in a dose-dependent manner. This is the case of rs2107538 and rs1924587. However, for other SNPs, the association was found confined either to the heterozygous or to the homozygous genotype. This could be linked to the low prevalence of the hetero/homozygous genotype in the study population. TNBC specific SNP **rs614367** was associated with prognosis in a BC subgroup-TNBC but not in all breast cancer patients. We also showed the functional implication of CCL5 breast cancer susceptibility locus and correlated the risk alleles of CCL5 SNPs with low levels of serum CCL5. Furthermore, we performed *in silico* analysis and found that low *CCL5* expression predicted a poor survival in BC and TNBC.

CCL5, which is originally known as RANTES (regulated on activation, normal T cell expressed and secreted), directs leukocytes to migrate into damaged or infected sites upon an inflammatory insult. In addition, CCL5 is essential to maintaining CD8 T cell function during chronic infection (41). It could be expected that high CCL5 levels at the tumor site would result in the recruitment of CD8^+^ T cells and sustain their cytotoxic capability against tumor cells. CCL5 expression is indeed associated with accumulation of CD8^+^ infiltrating T cells and associated with a favorable prognosis in colon cancer (42) and breast cancer (43). The risk allele of two SNPs in *CCL5* gene corresponds with a lower CCL5 expression, which leads to a depletion of CD8^+^ infiltrating T cells, and, impairment of CD8^+^ T cell function and thus a higher risk of malignancy. Nevertheless, we did not observe any significant association between *CCL5* polymorphism and survival or disease characteristics, except a suggestive effect of **rs2107538-T** allele on TNBC patient survival. This indicates that other type(s) of immune cells might play a dominant role in disease progression other than CD8^+^ T cells. Our *in silico* analyses, assessing the prognosis value of CCL5 expression, showed that low *CCL5* expression predicted a poor survival in BC and TNBC. These findings are in agreement with certain previous reports (43, 44) but contradict others (13). These discrepancies could be due to several reasons including the size of the study population. Unlike most of the previous studies, in the current study we performed analyses on large public datasets (34). Furthermore, we assessed the CCL5 expression in tumor specimen harboring both tumor and stroma cells which provides a more accurate assessment of the CCL5 effects not only on tumor cells but also on tumor microenvironment and tumor-infiltrating immune cells. Conversely, several previous reports limited their assessment of the effects of CCL5 expression on tumor cell behavior which could lead to biased conclusions.

Our study is limited by the relatively few deaths in patients which limited the power for survival analyses. To address this limitation, we analyzed TCGA data which strengthened our findings and revealed a significant association between the ***MAP3K21* rs1294255-G** allele and OVS in TNBC patients.

One of the goals of this study was to identify TNBC susceptibility SNPs given that immune pathways and activities were elevated in TNBC compared to non-TNBC. However, *CCL5* polymorphisms were not preferentially associated with TNBC, indicating that CCL5 also plays an important role in non-TNBC. CCL5 has been implicated in luminal type breast cancer by modulating the phenotype of CD4^+^ T cells (45) and in ER-positive breast cancers by promoting macrophage influx (46). The clinically established BC Intrinsic subtype classifiers, PAM50 (47) and claudin-low predictor (48), do not contain *CCL5* gene. All the above suggest a universal contribution of CCL5 in BC regardless of the subtypes. Tumor-derived CCL5 was detected in many clinical specimens and BC-derived cell lines, and were considered to promote BC aggressiveness (13). However, we and others showed that tumor-derived CCL5 expression did not significantly promote mammary carcinoma growth (10, 49), but the hematopoietic CCL5 played the major role in BC progression by regulating myeloid cells or macrophages in different subtype of BC (10, 50). Two recent studies suggested that intertumoral CCL5 level determined the recruitment of CD8^+^ T-cell in TNBC (43, 51). CCL5 might influence tumor growth by regulating different tumor-infiltrating immune cells in different subtypes of BC. Compared to ***CCL5* rs2107538**, the association of ***CCL5* rs2280788** and ***CCL5* rs2280789** was not that significant. However, the OR of the latter two are often relatively high even if only a slight significance or a suggestive significance. Unlike common variants with low penetrance, these two variants seem to have a medium penetrance in breast cancer.

We showed that ***CCND1* rs614367** was associated with a strong and specific increased risk for TNBC, and also had prognostic value only for TNBC. ***CCND1* rs614367** is one of the strongest breast cancer risk loci identified by GWAS studies; however, its specific association with TNBC has not been identified. Replication of these findings in other populations will be of interest to determine whether the association of the ***CCND1* rs614367** with TNBC can be generalized. In GWAS studies, **rs614367** was usually tagged to the ***CCND1*** gene, which encodes cyclin D1, a central component in the cell cycle that could be regulated by estrogen. Although *CCND1* is well-established as a cancer driver gene (52), it seems that *CCND1* is an ER-positive breast cancer marker (53). *FGF19* is one of its another nearby gene, and FGF19 and its receptor FGFR4 were strongly associated with the basal-like subtype of breast cancer (54). However, our *in-silico* analyses did not show any allele specific effect on these genes' expression. It will be of interest to further explore the functional implications of rs614367 like its potential long-range regulation or its association with non-coding RNA.

We also showed that ***HS6ST3* rs1924587** was preferentially associated with HRBC risk. HS6ST3 could promote breast cancer cell proliferation by upregulating IGF1R expression (29). It has been shown that IGF1R inhibits CCL5 expression and consequently reduces the chemotactic movement of CD8^+^ T lymphocytes (30). Taken together these findings suggest that through IGF1R, HS6ST3 could affect both CCL5 levels and tumor infiltrating CD8^+^ T lymphocytes behavior. This agrees with our finding showing that low levels of CCL5 expression associated with poor prognosis of BC. It is worth noting that IGF1R is abundant in HRBC but not in TNBC (55) which agrees with our current finding showing the preferential association between *HS6ST3* rs1924587 and HRBC risk and could also explain the predominant role of CCL5 in HRBC.

Analysis of multiple datasets indicates that high expression of *MAP3K21* is associated with a poor survival in breast cancer (56), and, the **C allele** of **rs1294255** is associated with a low expression of *MAP3K21* (Table S7). These findings are consistent with our analysis of the TCGA breast cancer dataset showing that **rs1294255-G** allele associated with poor prognosis. In the present study, we could not replicate the association, shown by GWAS, between **rs1294255** and TNBC risk, but rather we observed that the **rs1294255-C** allele was found more frequently in patients with no lymph-node metastasis resulting in a significantly negative OR associated with the heterozygous **rs1294255-CG**.

Although the GWAS studies in Caucasians and the meta-analysis could lead to the discovery of SNPs associated with breast cancer, discovery studies and independent validation in different population are needed given the ethnic specificity and environmental exposure. For example, TNBC is over-expressed in African-American populations (57) as well as Arab populations. However, the correlated gene expression data and genotyping data are not available for these two populations.

In conclusion, the results of this study suggest that genetic variation in *CCL5* and the *CCL5* signaling pathway might have not only susceptibility but also prognostic implications for TNBC. Furthermore, this work adds to the evidence that CCL5 and its mediators play a critical role in the process of TNBC development and aggressiveness.

## Data Availability Statement

The raw data supporting the conclusions of this manuscript will be made available by the authors, without undue reservation, to any qualified researcher.

## Ethics Statement

The studies involving human participants were reviewed and approved by Weill Cornell Medicine-Qatar IRB. The patients/participants provided their written informed consent to participate in this study.

## Author Contributions

SB, XM, DB, and LC contributed conception and design of the study. JS, AC, NB, YR, and SG performed the experiments. JS, YM, MS, RS, EZ, and JR analyzed the data. JS, MS, DB, and LC wrote the manuscript. All authors contributed to manuscript revision, read, and approved the submitted version.

### Conflict of Interest

The authors declare that the research was conducted in the absence of any commercial or financial relationships that could be construed as a potential conflict of interest.
