# Supplementary material for: Genetic Variation in CCL5 Signaling Genes and Triple Negative Breast Cancer: Susceptibility and Prognosis Implications
Source: Front Oncol. 2019 Dec 6;9:1328. doi: 10.3389/fonc.2019.01328 (PMC6915105; doi:10.3389/fonc.2019.01328)
Supplement: Supplementary file 1 [file Table_1.DOCX]

**Table S1** The description of 9 SNPs of the CCL5 and CCL5 signaling pathway

| **SNP** | **chr:locus (GRCh38)** | **Gene** | **Allele** | **1000G_EUR** | **HapMap_CEU** |
| --- | --- | --- | --- | --- | --- |
| rs2107538 | Chr.17: 35880776 | *CCL5* | C>T | 0.16 | 0.16 |
| rs2280788 | Chr.17: 35880401 | *CCL5* | C>G | 0.01 | 0.01 |
| rs2280789 | Chr.17: 35879999 | *CCL5* | A>G | 0.11 | 0.10 |
| rs614367 | Chr.11: 69513996 | *CCND1* | C>T | -* | 0.19 |
| rs704010 | Chr.10: 79081391 | *ZMIZ1* | C>T | 0.41 | 0.43 |
| rs1045485 | Chr.2: 201284866 | *CASP8* | G>C | 0.12 | 0.13 |
| rs1124933 | Chr.20: 18097908 | *NOTCH2* | G>A | 0.41 | 0.45 |
| rs1294255 | Chr.1: 233365612 | *MAP3K21* | G>C | 0.39 | 0.44 |
| rs1924587 | Chr.13: 96745125 | *HS6ST3* | G>C | 0.42 | 0.51 |

* Data is not available in 1000 Genome dataset
