# Supplementary material for: Genetic Variation in CCL5 Signaling Genes and Triple Negative Breast Cancer: Susceptibility and Prognosis Implications
Source: Front Oncol. 2019 Dec 6;9:1328. doi: 10.3389/fonc.2019.01328 (PMC6915105; doi:10.3389/fonc.2019.01328)
Supplement: Supplementary file 2 [file Table_2.DOCX]

**Table S2** Genotype distributions in cases and controls

| **Genotype** | **Cases (N=544)** | | **HWE*** | **Controls (N=538)** | | **HWE** |
| --- | --- | --- | --- | --- | --- | --- |
|  | **N** | **Frequency** |  | **N** | **Frequency** |  |
| rs2107538 |  |  |  |  |  |  |
| CC | 349 | 0.641 | 0.251 | 389 | 0.723 | 0.760 |
| CT | 168 | 0.309 |  | 138 | 0.257 |  |
| TT | 27 | 0.050 |  | 11 | 0.020 |  |
| rs2280788 |  |  |  |  |  |  |
| GG | 535 | 0.983 | 0.000007 | 535 | 0.994 | 0.948 |
| GC | 8 | 0.015 |  | 3 | 0.006 |  |
| CC | 1 | 0.002 |  | 0 | 0.000 |  |
| rs2280789 |  |  |  |  |  |  |
| AA | 410 | 0.754 | 0.861 | 423 | 0.786 | 0.052 |
| AG | 124 | 0.228 |  | 113 | 0.210 |  |
| GG | 10 | 0.018 |  | 2 | 0.004 |  |
| rs614367 |  |  |  |  |  |  |
| CC | 418 | 0.768 | 0.525 | 435 | 0.809 | 0.056 |
| CT | 112 | 0.206 |  | 99 | 0.184 |  |
| TT | 14 | 0.026 |  | 4 | 0.007 |  |
| rs704010 |  |  |  |  |  |  |
| CC | 262 | 0.482 | 0.100 | 271 | 0.504 | 0.727 |
| CT | 219 | 0.403 |  | 224 | 0.416 |  |
| TT | 63 | 0.116 |  | 43 | 0.080 |  |
| rs1045485 |  |  |  |  |  |  |
| GG | 413 | 0.759 | 0.055 | 416 | 0.773 | 0.269 |
| GC | 116 | 0.213 |  | 111 | 0.206 |  |
| CC | 15 | 0.028 |  | 11 | 0.020 |  |
| rs1124933 |  |  |  |  |  |  |
| GG | 262 | 0.482 | 0.889 | 233 | 0.433 | 0.056 |
| GA | 217 | 0.399 |  | 241 | 0.448 |  |
| AA | 65 | 0.119 |  | 64 | 0.119 |  |
| rs1294255 |  |  |  |  |  |  |
| GG | 191 | 0.351 | 0.056 | 192 | 0.357 | 0.148 |
| GC | 244 | 0.449 |  | 245 | 0.455 |  |
| CC | 109 | 0.200 |  | 101 | 0.188 |  |
| rs1924587 |  |  |  |  |  |  |
| GG | 178 | 0.327 | 0.948 | 216 | 0.401 | 0.430 |
| GC | 267 | 0.491 |  | 243 | 0.452 |  |
| CC | 99 | 0.182 |  | 79 | 0.147 |  |

**P* value is calculated by Pearson's chi-square
