# Supplementary material for: Genetic Variation in CCL5 Signaling Genes and Triple Negative Breast Cancer: Susceptibility and Prognosis Implications
Source: Front Oncol. 2019 Dec 6;9:1328. doi: 10.3389/fonc.2019.01328 (PMC6915105; doi:10.3389/fonc.2019.01328)
Supplement: Supplementary file 3 [file Table_3.DOCX]

**Table S3** Association of 9 SNPs with Hormone Receptor Positive Breast Cancer Risk

| **SNP** | **Genotype** | **Controls** | **Cases** | **Heterozygotes** | |  | **Homozygotes** | |  | **Per Risk Allele** | |
| --- | --- | --- | --- | --- | --- | --- | --- | --- | --- | --- | --- |
|  |  | ***N*=538** | ***N*=196** | **OR (95%CI)** | ***P*** |  | **OR (95%CI)** | ***P*** |  | **OR (95%CI)** | ***P*** |
|  |  |  |  |  |  |  |  |  |  |  |  |
| rs2107538 | CC | 389 | 190 | **1.78(1.24-2.54)** | **0.002**** |  | **3.39(1.19-9.68)** | **0.016**** |  | **1.8(1.33-2.44)** | **0.0001** |
|  | CT | 138 | 98 |  |  |  |  |  |  |  |  |
|  | TT | 11 | 13 |  |  |  |  |  |  |  |  |
| rs2280788 | GG | 535 | 296 | 3.23(0.59-17.74) | NS |  | 4.84(0.2-119.24) | NS |  | 4.82(0.97-23.97) | 0.065 |
|  | GC | 3 | 4 |  |  |  |  |  |  |  |  |
|  | CC | 0 | 1 |  |  |  |  |  |  |  |  |
| rs2280789 | AA | 423 | 222 | 1.29(0.92-1.82) | NS |  | 4.28(0.82-22.24) | NS |  | **1.36(1.0-1.86)** | **0.048** |
|  | AG | 113 | 74 |  |  |  |  |  |  |  |  |
|  | GG | 2 | 5 |  |  |  |  |  |  |  |  |
| rs614367 | CC | 435 | 241 | 0.985(0.68-1.42) | NS |  | 2.71(0.76-9.69) | NS |  | 1.12(0.81-1.54) | NS |
|  | CT | 99 | 54 |  |  |  |  |  |  |  |  |
|  | TT | 4 | 6 |  |  |  |  |  |  |  |  |
| rs704010 | CC | 271 | 146 | 0.98(0.72-1.32) | NS |  | 1.60(0.99-2.59) | 0.056* |  | 1.16(0.93-1.44) | NS |
|  | CT | 224 | 118 |  |  |  |  |  |  |  |  |
|  | TT | 43 | 37 |  |  |  |  |  |  |  |  |
| rs1045485 | GG | 416 | 234 | 0.87(0.6-1.24) | NS |  | 2.1(0.93-4.76) | NS |  | 1.09(0.81-1.46) | NS |
|  | GC | 111 | 54 |  |  |  |  |  |  |  |  |
|  | CC | 11 | 13 |  |  |  |  |  |  |  |  |
| rs1124933 | GG | 233 | 143 | 0.86(0.64-1.16) | NS |  | 0.79(0.49-1.27) | NS |  | 0.88(0.71-1.09) | NS |
|  | GA | 241 | 127 |  |  |  |  |  |  |  |  |
|  | AA | 64 | 31 |  |  |  |  |  |  |  |  |
| rs1294255 | GG | 192 | 103 | 1.04(0.76-1.43) | NS |  | 1.1(0.74-1.63) | NS |  | 1.1(074-1.63) | NS |
|  | GC | 245 | 138 |  |  |  |  |  |  |  |  |
|  | CC | 101 | 60 |  |  |  |  |  |  |  |  |
| rs1924587 | GG | 216 | 95 | 1.37(0.99-1.9) | 0.057* |  | **1.7(1.12-2.57)** | **0.012**** |  | **1.33(1.08-1.64)** | **0.006** |
|  | GC | 243 | 142 |  |  |  |  |  |  |  |  |
|  | CC | 79 | 64 |  |  |  |  |  |  |  |  |

CI confidence interval, OR odds ratio

*P* value was obtained by χ2 test. Significant *P* values are in bold cases.

** The association remains significant after age and menopausal status adjustment

* Not significant after age and menopausal status adjustment
