# Supplementary material for: Genetic Variation in CCL5 Signaling Genes and Triple Negative Breast Cancer: Susceptibility and Prognosis Implications
Source: Front Oncol. 2019 Dec 6;9:1328. doi: 10.3389/fonc.2019.01328 (PMC6915105; doi:10.3389/fonc.2019.01328)
Supplement: Supplementary file 4 [file Table_4.DOCX]

**Table S4** Clinicopathologic characteristics of breast cancer patients and the corresponding univariate analysis of death (Progression–free survival, PFS)

| **Characteristic** | **%** | **Breast cancer specific PFS** | |
| --- | --- | --- | --- |
|  |  | **6-year rate** | ***P*** |
| Tumor Size |  |  |  |
| T_0-2_ | 67.8 | 82.1 | **0.002** |
| T_3-4_ | 32.2 | 69.8 |  |
| Regional lymph node status | |  |  |
| N_0_ | 60.3 | 80.0 | 0.063 |
| N_1-3_ | 39.7 | 77.7 |  |
| Pathological lymph node stage | |  |  |
| pN_0_ | 41.5 | 83.0 | **0.015** |
| pN_1-3_ | 58.5 | 75.0 |  |
| Tumor Grade |  |  |  |
| SBR_1-2_ | 64.6 | 79.9 | **0.038** |
| SBR_3_ | 35.4 | 71.8 |  |
| age |  |  |  |
| <50 | 60.3 | 79.8 | NS |
| ≥50 | 39.7 | 77.5 |  |
