# Supplementary material for: Genetic Variation in CCL5 Signaling Genes and Triple Negative Breast Cancer: Susceptibility and Prognosis Implications
Source: Front Oncol. 2019 Dec 6;9:1328. doi: 10.3389/fonc.2019.01328 (PMC6915105; doi:10.3389/fonc.2019.01328)
Supplement: Supplementary file 5 [file Table_5.DOCX]

**Table S5** Association between 9 SNPs and clinicopathologic characteristics

| SNP | Genotype | Tumor size | | |  | Regional lymph nodes | | |  | Pathological lymph node stage | | |  | Tumor grade | | |  | Distant metastasis | | |  | Relapse | | |
| --- | --- | --- | --- | --- | --- | --- | --- | --- | --- | --- | --- | --- | --- | --- | --- | --- | --- | --- | --- | --- | --- | --- | --- | --- |
|  |  | T_0-2_ | T_3-4_ | *P* |  | N_0_ | N_1-3_ | *P* |  | pN_0_ | pN_1-3_ | *P* |  | SBR_1-2_ | SBR_3_ | *P* |  | M_0_ | M_1_ | *P* |  | 0 | 1 | *P* |
| rs2107538 | CC | 232 | 88 | 0.082 |  | 201 | 126 | NS |  | 124 | 203 | NS |  | 206 | 106 | NS |  | 294 | 55 | NS |  | 287 | 61 | NS |
|  | CT | 105 | 58 |  |  | 87 | 74 |  |  | 55 | 106 |  |  | 100 | 55 |  |  | 150 | 18 |  |  | 146 | 22 |  |
|  | TT | 15 | 11 |  |  | 18 | 8 |  |  | 11 | 14 |  |  | 16 | 6 |  |  | 21 | 6 |  |  | 22 | 5 |  |
|  |  |  |  |  |  |  |  |  |  |  |  |  |  |  |  |  |  |  |  |  |  |  |  |  |
| rs2280788 | GG | 347 | 153 | NS |  | 300 | 205 | NS |  | 186 | 318 | NS |  | 316 | 169 | NS |  | 457 | 78 | NS |  | 446 | 88 | NS |
|  | GC | 5 | 3 |  |  | 5 | 3 |  |  | 4 | 4 |  |  | 5 | 3 |  |  | 7 | 1 |  |  | 8 | 0 |  |
|  | CC | 1 | 0 |  |  | 1 | 0 |  |  | 0 | 1 |  |  | 1 | 0 |  |  | 1 | 0 |  |  | 1 | 0 |  |
|  |  |  |  |  |  |  |  |  |  |  |  |  |  |  |  |  |  |  |  |  |  |  |  |  |
| rs2280789 | AA | 269 | 112 | NS |  | 231 | 155 | NS |  | 147 | 240 | NS |  | 237 | 130 | NS |  | 349 | 61 | NS |  | 341 | 68 | NS |
|  | AG | 79 | 42 |  |  | 69 | 49 |  |  | 39 | 77 |  |  | 77 | 41 |  |  | 108 | 16 |  |  | 106 | 18 |  |
|  | GG | 6 | 3 |  |  | 6 | 4 |  |  | 4 | 6 |  |  | 7 | 1 |  |  | 8 | 2 |  |  | 8 | 2 |  |
|  |  |  |  |  |  |  |  |  |  |  |  |  |  |  |  |  |  |  |  |  |  |  |  |  |
| rs614367 | CC | 285 | 115 | NS |  | 246 | 158 | NS |  | 152 | 252 | NS |  | 250 | 136 | NS |  | 368 | 61 | NS |  | 363 | 65 | NS |
|  | CT | 58 | 38 |  |  | 52 | 43 |  |  | 32 | 62 |  |  | 59 | 34 |  |  | 84 | 16 |  |  | 81 | 19 |  |
|  | TT | 11 | 4 |  |  | 8 | 7 |  |  | 6 | 9 |  |  | 13 | 2 |  |  | 13 | 2 |  |  | 11 | 4 |  |
|  |  |  |  |  |  |  |  |  |  |  |  |  |  |  |  |  |  |  |  |  |  |  |  |  |
| rs704010 | CC | 174 | 76 | NS |  | 147 | 102 | NS |  | 89 | 153 | NS |  | 159 | 78 | NS |  | 222 | 40 | NS |  | 220 | 41 | NS |
|  | CT | 142 | 62 |  |  | 128 | 77 |  |  | 78 | 132 |  |  | 129 | 74 |  |  | 190 | 29 |  |  | 183 | 36 |  |
|  | TT | 40 | 19 |  |  | 31 | 29 |  |  | 23 | 38 |  |  | 34 | 19 |  |  | 53 | 10 |  |  | 52 | 11 |  |
|  |  |  |  |  |  |  |  |  |  |  |  |  |  |  |  |  |  |  |  |  |  |  |  |  |
| rs1045485 | GG | 277 | 128 | NS |  | 239 | 166 | NS |  | 148 | 259 | NS |  | 257 | 138 | NS |  | 364 | 66 | NS |  | 353 | 76 | NS |
|  | GC | 59 | 23 |  |  | 51 | 33 |  |  | 36 | 46 |  |  | 48 | 28 |  |  | 78 | 11 |  |  | 78 | 11 |  |
|  | CC | 18 | 6 |  |  | 16 | 9 |  |  | 6 | 18 |  |  | 17 | 6 |  |  | 23 | 2 |  |  | 24 | 1 |  |
|  |  |  |  |  |  |  |  |  |  |  |  |  |  |  |  |  |  |  |  |  |  |  |  |  |
| rs1124933 | GG | 165 | 77 | NS |  | 140 | 102 | NS |  | 85 | 157 | NS |  | 153 | 82 | NS |  | 231 | 30 | NS |  | 227 | 33 | 0.088 |
|  | GA | 141 | 65 |  |  | 124 | 94 |  |  | 77 | 133 |  |  | 128 | 68 |  |  | 180 | 37 |  |  | 176 | 41 |  |
|  | AA | 47 | 15 |  |  | 41 | 22 |  |  | 28 | 33 |  |  | 39 | 21 |  |  | 53 | 12 |  |  | 51 | 14 |  |
|  |  |  |  |  |  |  |  |  |  |  |  |  |  |  |  |  |  |  |  |  |  |  |  |  |
| rs1294255 | GG | 126 | 50 | NS |  | 104 | 75 | **0.005** |  | 69 | 116 | **0.046** |  | 102 | 71 | 0.079 |  | 160 | 31 | NS |  | 155 | 36 | NS |
|  | GC | 158 | 74 |  |  | 152 | 78 |  |  | 95 | 135 |  |  | 155 | 67 |  |  | 208 | 36 |  |  | 204 | 39 |  |
|  | CC | 70 | 33 |  |  | 50 | 55 |  |  | 26 | 71 |  |  | 65 | 34 |  |  | 97 | 12 |  |  | 96 | 13 |  |
|  |  |  |  |  |  |  |  |  |  |  |  |  |  |  |  |  |  |  |  |  |  |  |  |  |
| rs1924587 | GG | 120 | 45 | NS |  | 100 | 70 | NS |  | 71 | 99 | NS |  | 108 | 53 | NS |  | 155 | 22 | NS |  | 150 | 28 | NS |
|  | GC | 170 | 84 |  |  | 146 | 108 |  |  | 87 | 159 |  |  | 153 | 93 |  |  | 224 | 43 |  |  | 222 | 45 |  |
|  | CC | 64 | 28 |  |  | 60 | 30 |  |  | 32 | 65 |  |  | 61 | 26 |  |  | 86 | 13 |  |  | 83 | 15 |  |
