# Supplementary material for: Genetic Variation in CCL5 Signaling Genes and Triple Negative Breast Cancer: Susceptibility and Prognosis Implications
Source: Front Oncol. 2019 Dec 6;9:1328. doi: 10.3389/fonc.2019.01328 (PMC6915105; doi:10.3389/fonc.2019.01328)
Supplement: Supplementary file 6 [file Table_6.DOCX]

**Table S6** Association between *MAP3K21* rs1294255 and lymph node invasion

| **Genotype** | **Regional lymph nodes** | | | |  | | **Pathological lymph node stage** | | | | |  |
| --- | --- | --- | --- | --- | --- | --- | --- | --- | --- | --- | --- | --- |
|  | **N_0_** | **N_1-3_** | **OR(95%CI)** | ***P*** | |  | | **pN_0_** | **pN_1-3_** | **OR(95%CI)** | ***P*** | |
| CC | 50 | 55 | 1.00 |  | |  | | 26 | 71 | 1.00 |  | |
| GC | 152 | 78 | **0.47(0.29-0.74)** | **0.001** | |  | | 95 | 135 | **0.52(0.31-0.88)** | **0.013** | |
| GG | 104 | 75 | 0.66(0.40-1.06) | 0.087 | |  | | 69 | 116 | 0.61(0.36-1.06) | 0.077 | |

CI confidence interval, OR odds ratio

*P* value was obtained by χ2 test. The lymph node status was dichotomized as follows: nodal status ≥1 vs. no positive lymph node. Significant *P* values are in bold cases.
