# Supplementary material for: Genetic Variation in CCL5 Signaling Genes and Triple Negative Breast Cancer: Susceptibility and Prognosis Implications
Source: Front Oncol. 2019 Dec 6;9:1328. doi: 10.3389/fonc.2019.01328 (PMC6915105; doi:10.3389/fonc.2019.01328)
Supplement: Supplementary file 7 [file Table_7.DOCX]

**Table S7** NESDA NTR eQTL analysis of 9 SNPs with their tagged genes

| **SNP** | **Probeset ID** | **Gene** | **Beta** | ***P*-value** | **FDR** |
| --- | --- | --- | --- | --- | --- |
| rs2107538 | 11753810_a_at | *CCL5* | -0.133 | 1.39E-11 | <1.34e-05 |
| rs2107538 | 11732276_x_at | *CCL5* | -0.113 | 1.24E-08 | 1.26E-04 |
| rs2107538 | 11732275_at | *CCL5* | -0.116 | 1.88E-08 | 1.76E-04 |
| rs1294255 | 11724995_a_at | *MAP3K21* | -0.156 | 2.52E-15 | <1.34e-05 |
