# Supplementary figures and images for: Genetic Variation in CCL5 Signaling Genes and Triple Negative Breast Cancer: Susceptibility and Prognosis Implications
Source: Front Oncol. 2019 Dec 6;9:1328. doi: 10.3389/fonc.2019.01328 (PMC6915105; doi:10.3389/fonc.2019.01328)

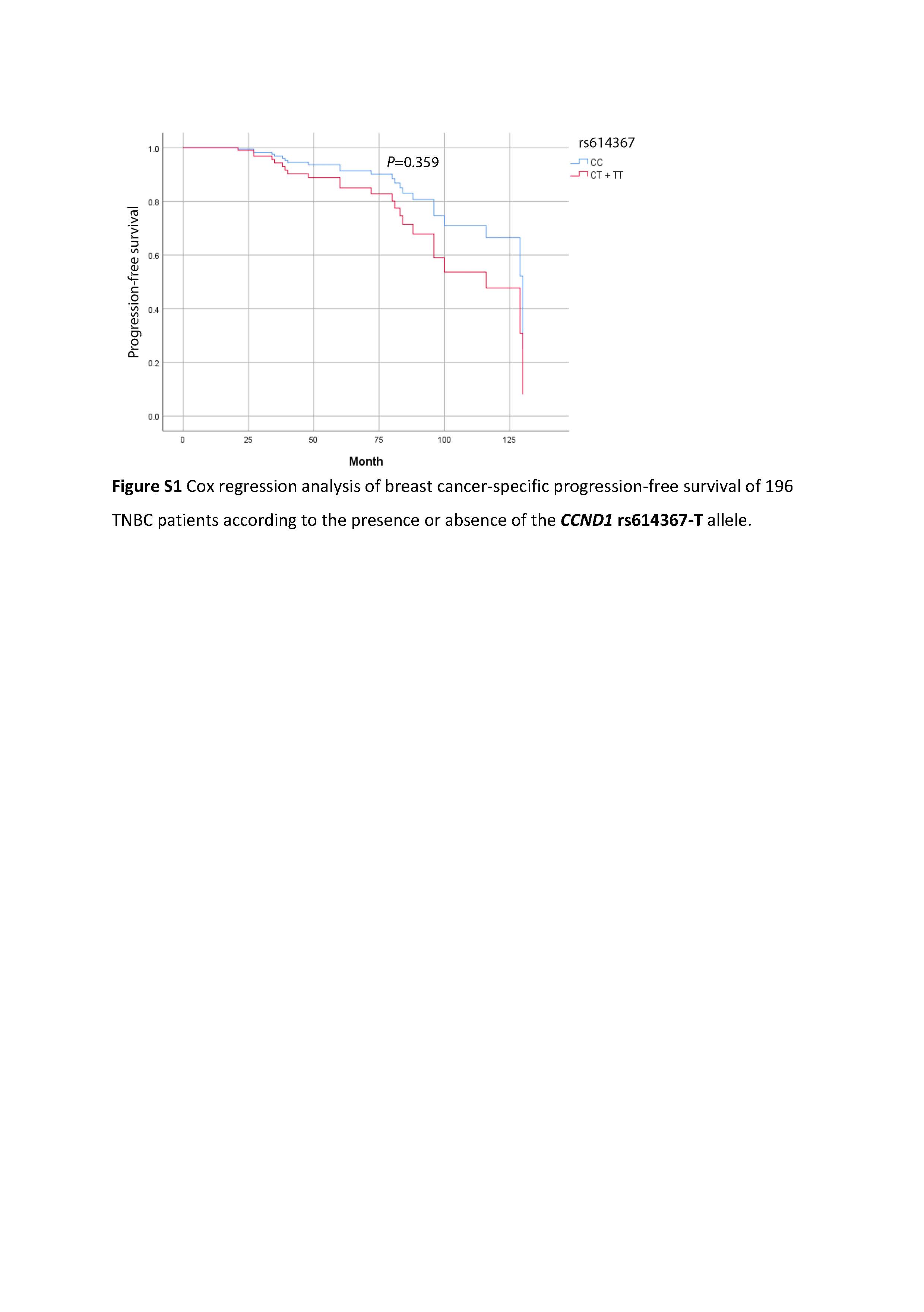

Supplement: Supplementary file 8 [file Image_1.JPEG]

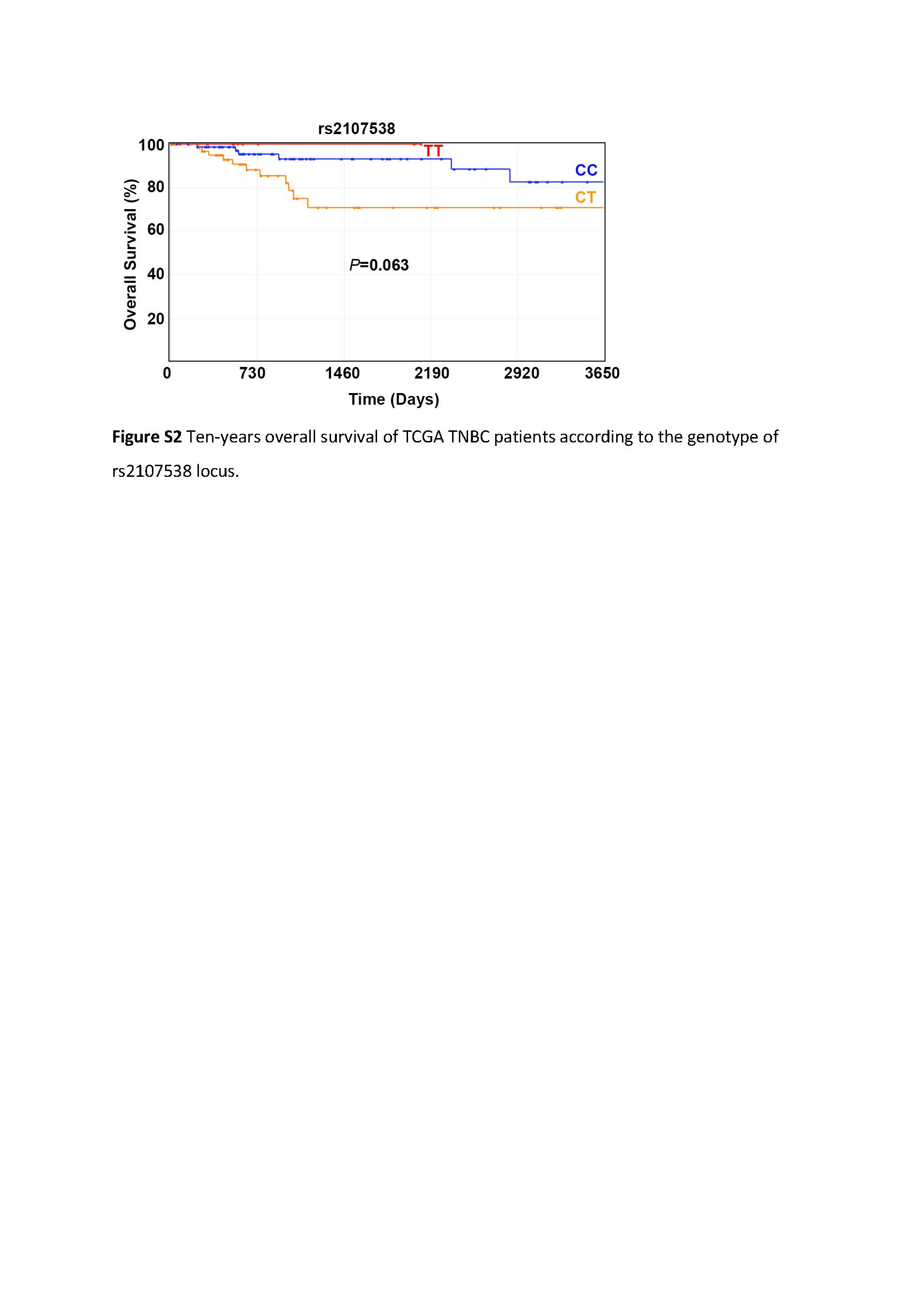

Supplement: Supplementary file 9 [file Image_2.JPEG]

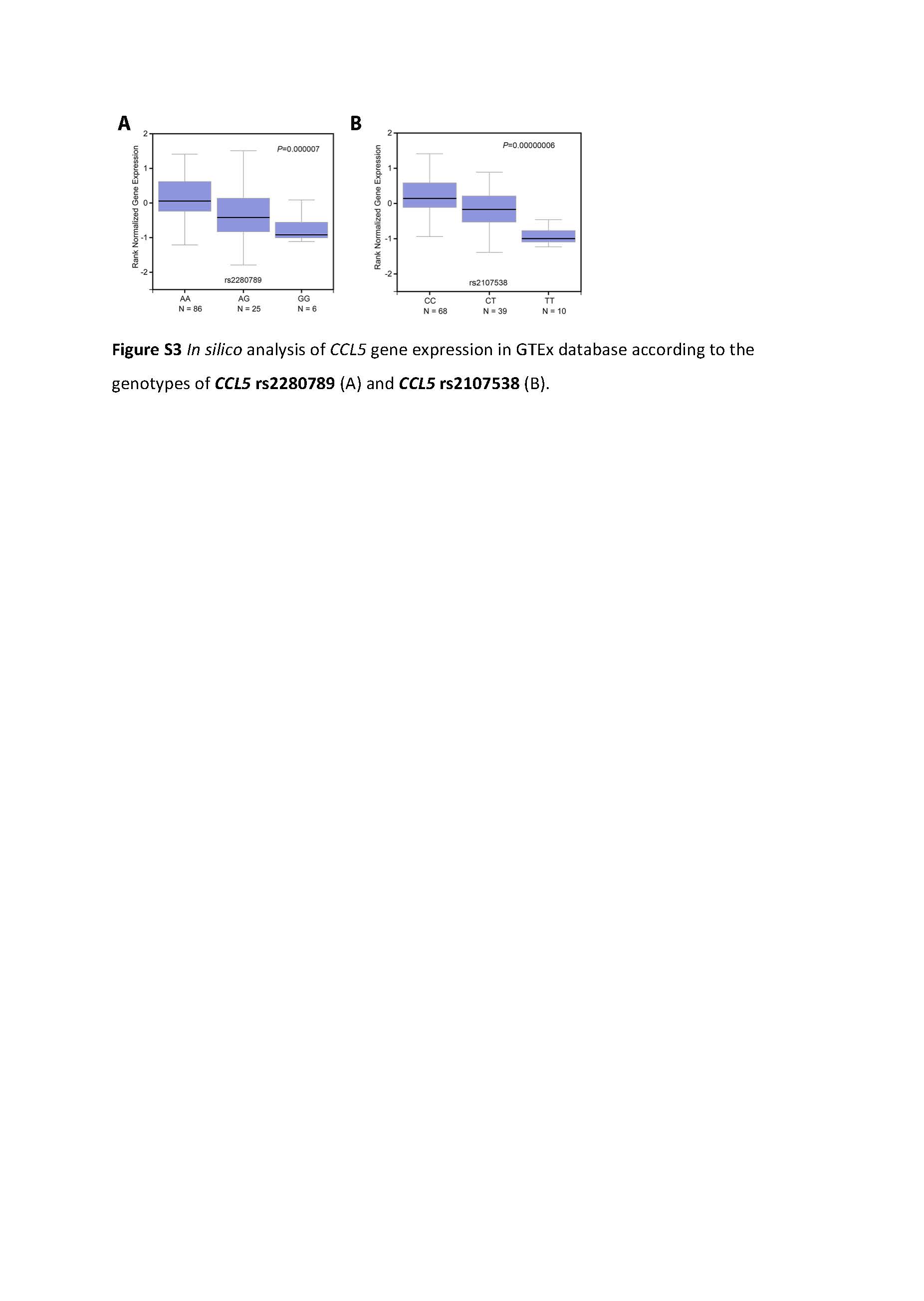

Supplement: Supplementary file 10 [file Image_3.JPEG]

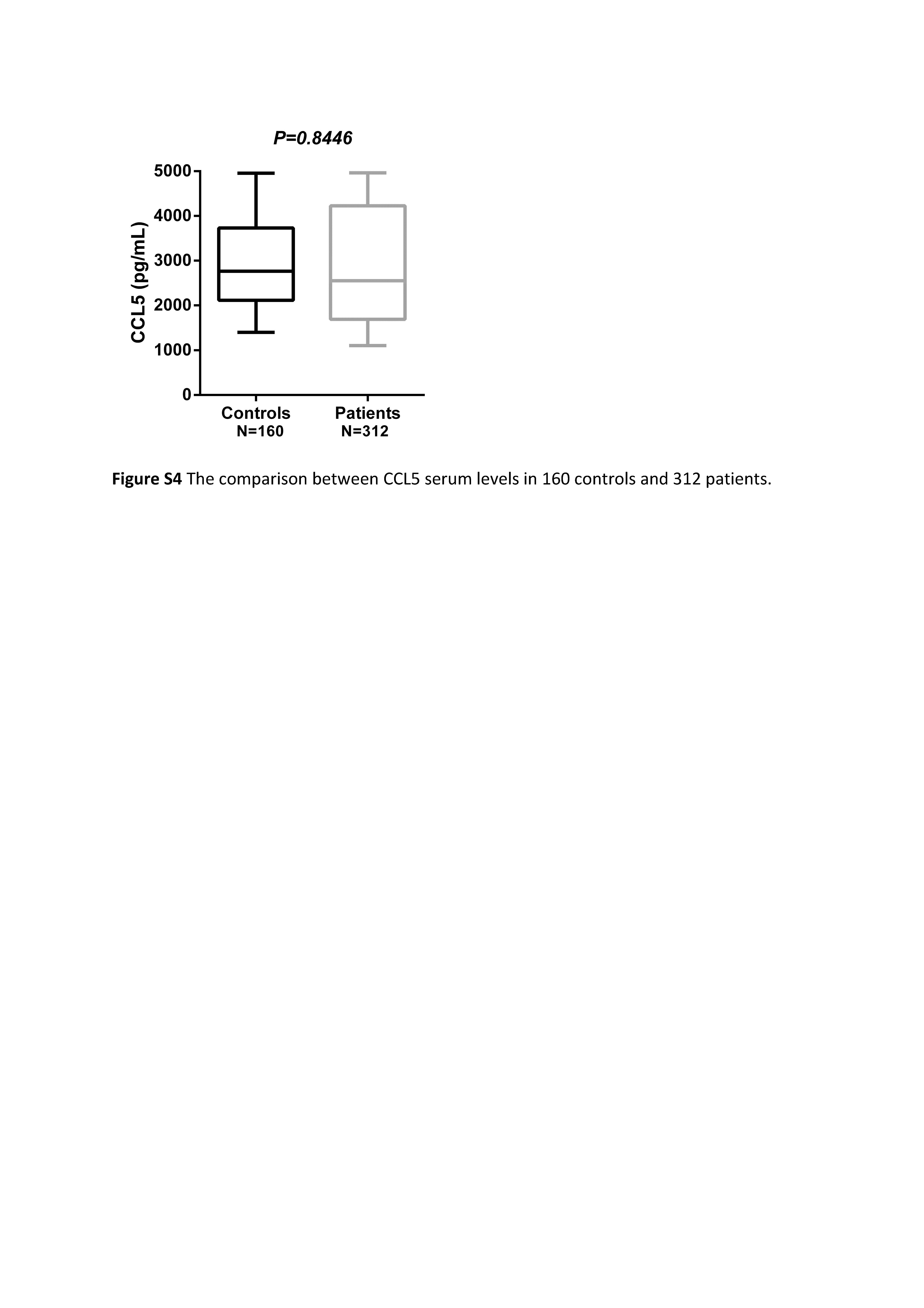

Supplement: Supplementary file 11 [file Image_4.JPEG]

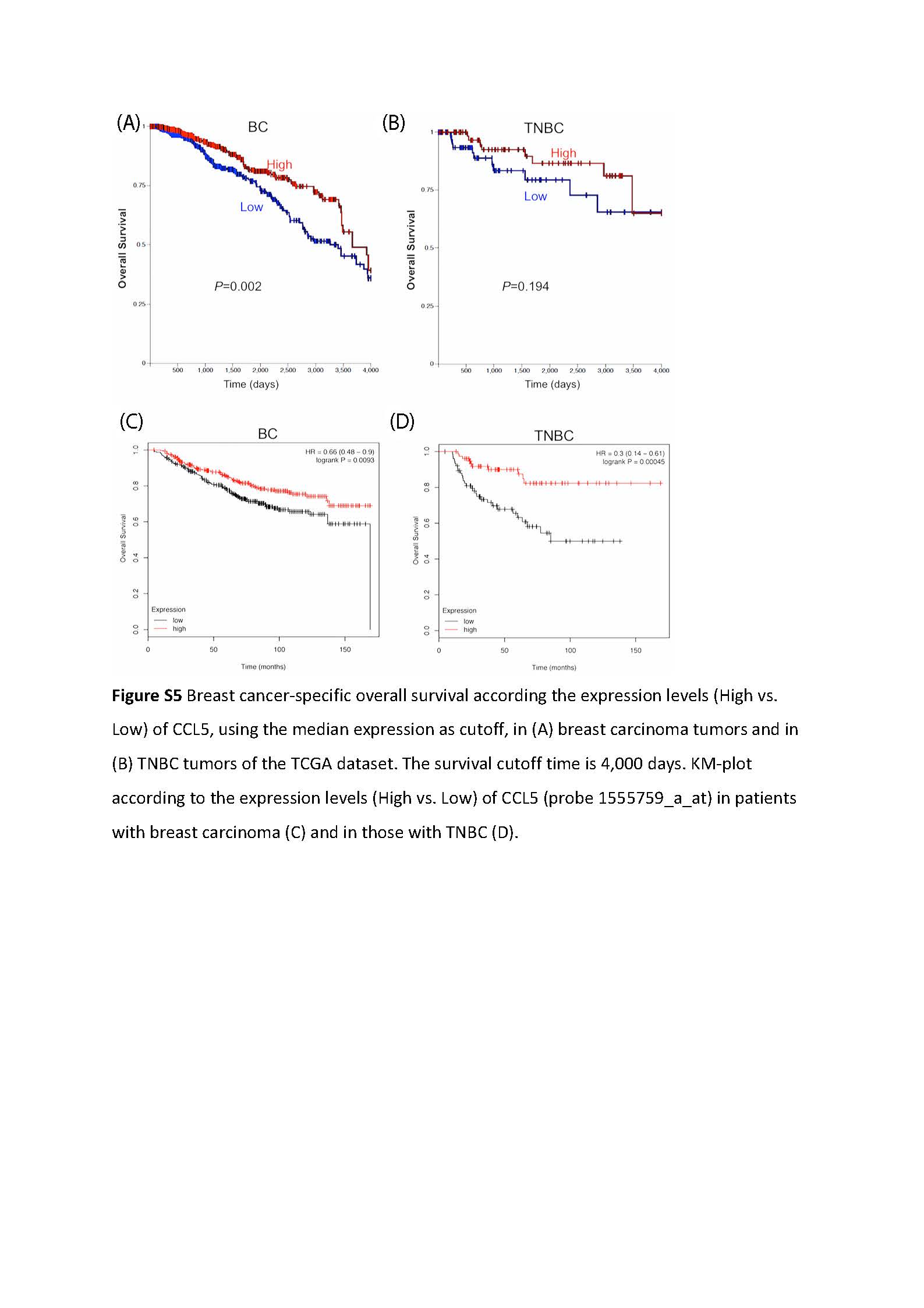

Supplement: Supplementary file 12 [file Image_5.JPEG]

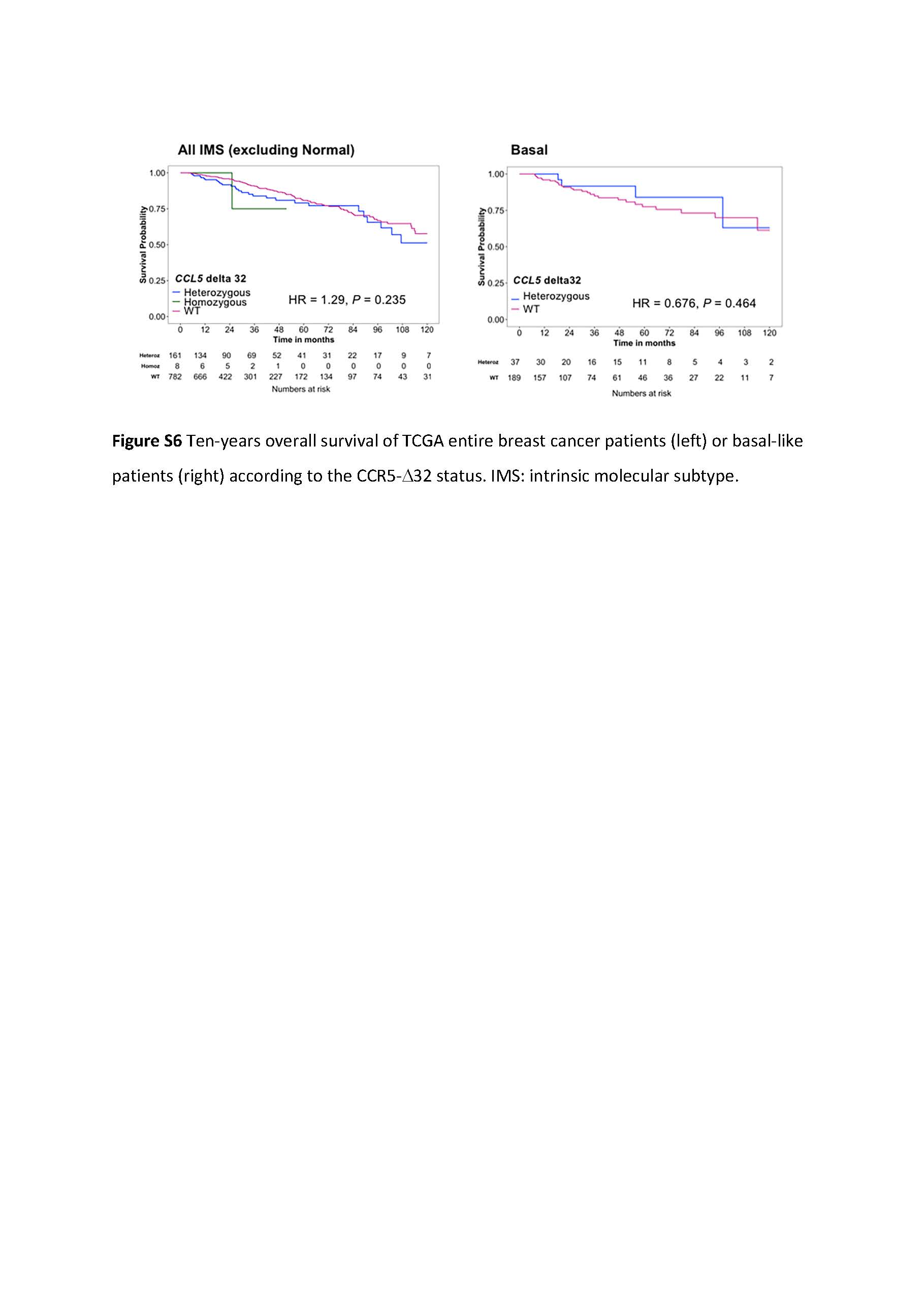

Supplement: Supplementary file 13 [file Image_6.JPEG]
